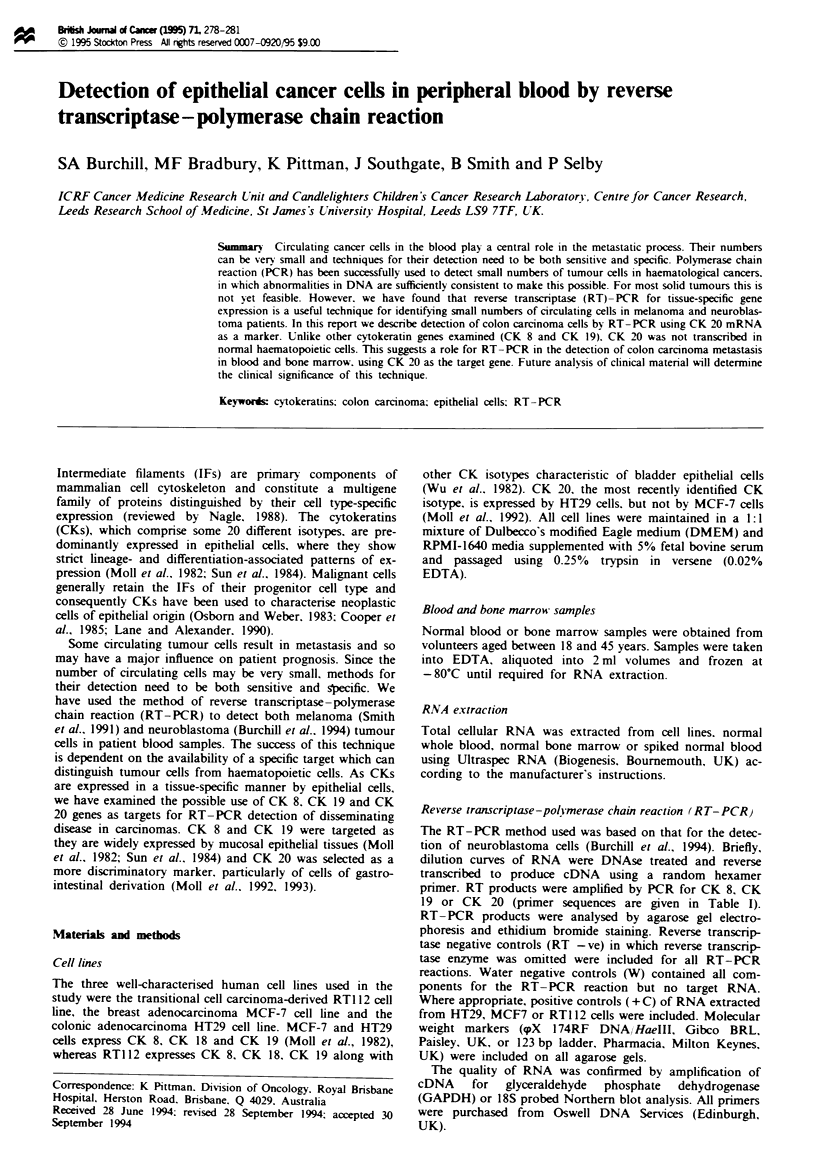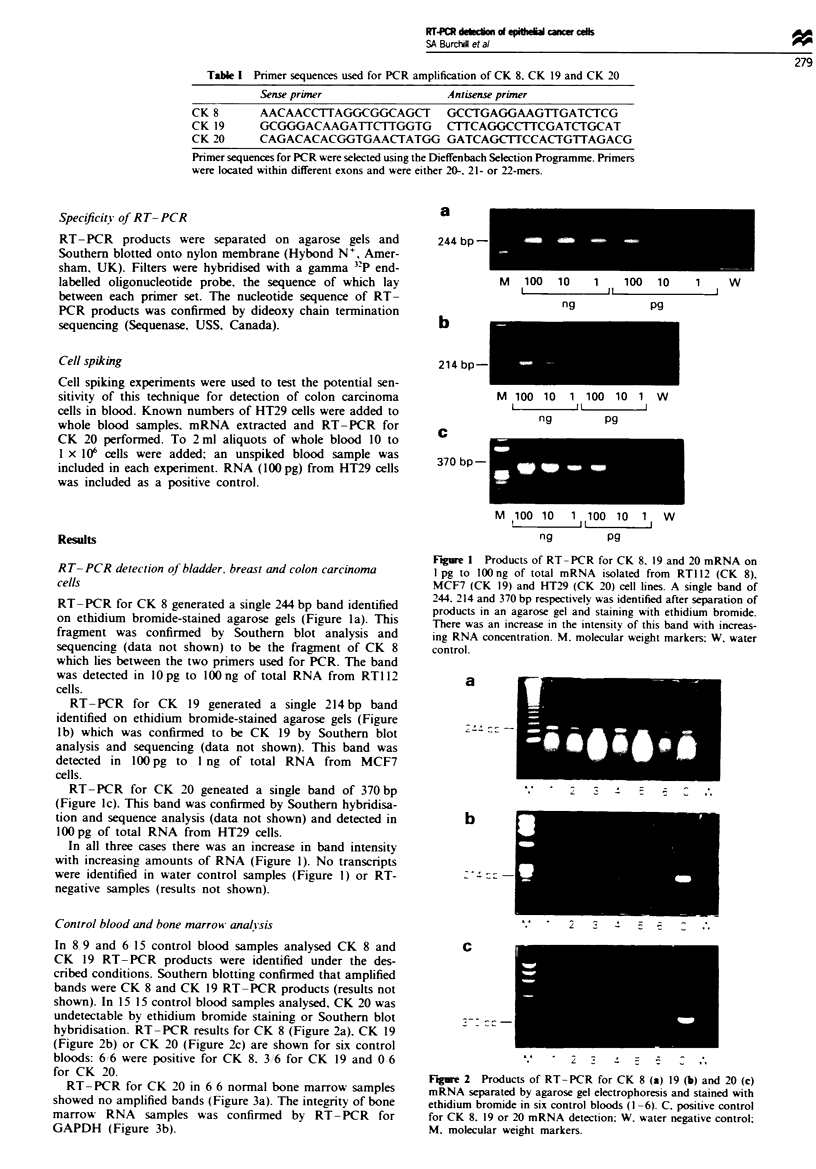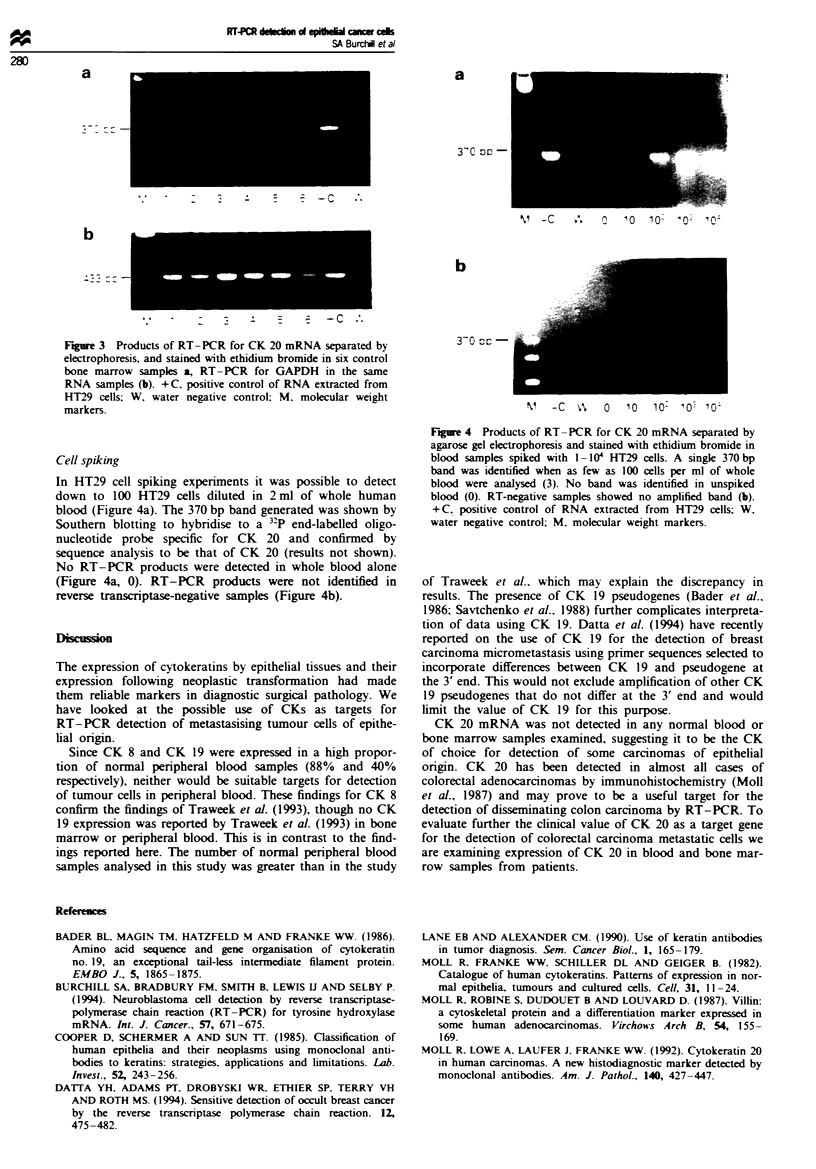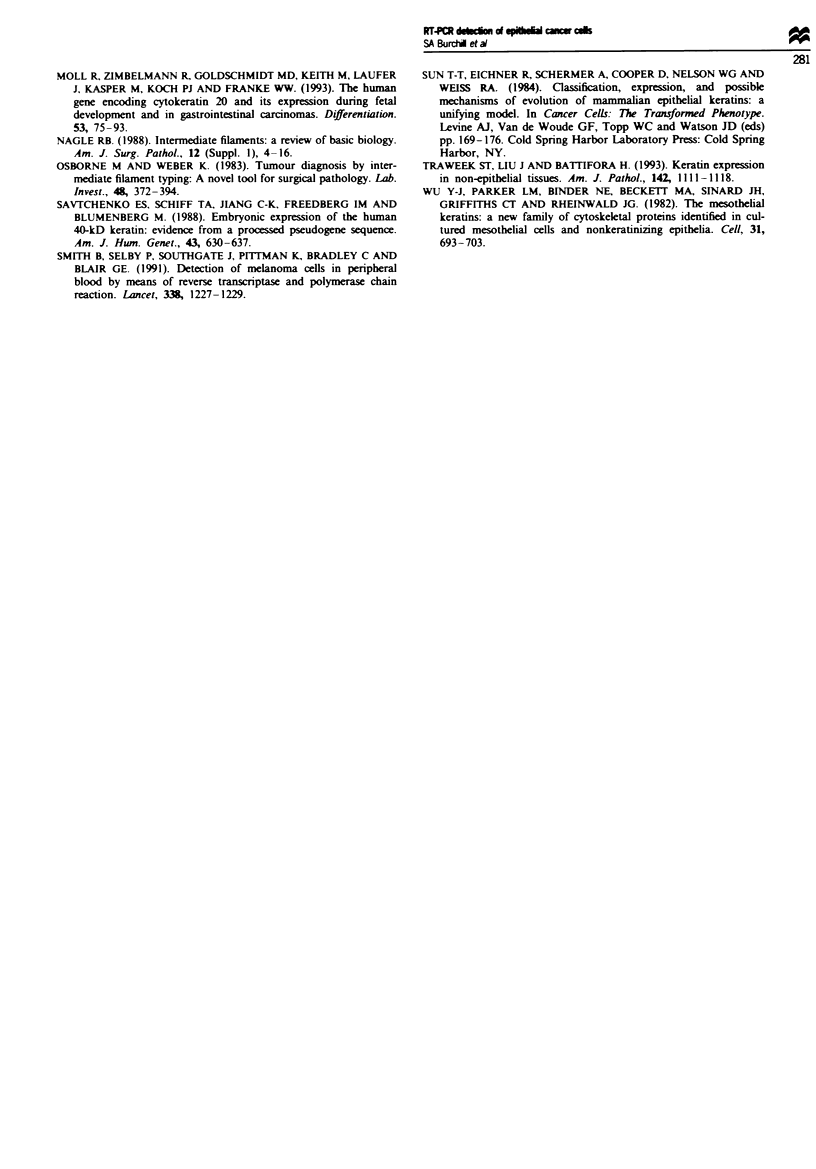# Detection of epithelial cancer cells in peripheral blood by reverse transcriptase-polymerase chain reaction.

**DOI:** 10.1038/bjc.1995.56

**Published:** 1995-02

**Authors:** S. A. Burchill, M. F. Bradbury, K. Pittman, J. Southgate, B. Smith, P. Selby

**Affiliations:** ICRF Cancer Medicine Research Unit, Leeds Research School of Medicine, St. James's University Hospital, UK.

## Abstract

**Images:**


British Journal of Cancer (1995) 71, 278-281

© 1995 Stockton Press All rights reserved 0007-0920/95 $9.00

### Detection of epithelial cancer cells in peripheral blood by reverse transcriptase–polymerase chain reaction

SA Burchill, MF Bradbury, K Pittman, J Southgate, B Smith and P Selby

*ICRF Cancer Medicine Research Unit and Candlelighters Children's Cancer Research Laboratory, Centre for Cancer Research, Leeds Research School of Medicine, St James's University Hospital, Leeds LS9 7TF, UK.*

**Summary** Circulating cancer cells in the blood play a central role in the metastatic process. Their numbers can be very small and techniques for their detection need to be both sensitive and specific. Polymerase chain reaction (PCR) has been successfully used to detect small numbers of tumour cells in haematological cancers, in which abnormalities in DNA are sufficiently consistent to make this possible. For most solid tumours this is not yet feasible. However, we have found that reverse transcriptase (RT)-PCR for tissue-specific gene expression is a useful technique for identifying small numbers of circulating cells in melanoma and neuroblastoma patients. In this report we describe detection of colon carcinoma cells by RT-PCR using CK 20 mRNA as a marker. Unlike other cytokeratin genes examined (CK 8 and CK 19), CK 20 was not transcribed in normal haematopoietic cells. This suggests a role for RT-PCR in the detection of colon carcinoma metastasis in blood and bone marrow, using CK 20 as the target gene. Future analysis of clinical material will determine the clinical significance of this technique.

**Keywords:** cytokeratins; colon carcinoma; epithelial cells; RT-PCR

Intermediate filaments (IFs) are primary components of mammalian cell cytoskeleton and constitute a multigene family of proteins distinguished by their cell type-specific expression (reviewed by Nagle, 1988). The cytokeratins (CKs), which comprise some 20 different isotypes, are predominantly expressed in epithelial cells, where they show strict lineage- and differentiation-associated patterns of expression (Moll *et al*., 1982; Sun *et al*., 1984). Malignant cells generally retain the IFs of their progenitor cell type and consequently CKs have been used to characterise neoplastic cells of epithelial origin (Osborn and Weber, 1983; Cooper *et al*., 1985; Lane and Alexander, 1990).

Some circulating tumour cells result in metastasis and so may have a major influence on patient prognosis. Since the number of circulating cells may be very small, methods for their detection need to be both sensitive and specific. We have used the method of reverse transcriptase-polymerase chain reaction (RT-PCR) to detect both melanoma (Smith *et al*., 1991) and neuroblastoma (Burchill *et al*., 1994) tumour cells in patient blood samples. The success of this technique is dependent on the availability of a specific target which can distinguish tumour cells from haematopoietic cells. As CKs are expressed in a tissue-specific manner by epithelial cells, we have examined the possible use of CK 8, CK 19 and CK 20 genes as targets for RT-PCR detection of disseminating disease in carcinomas. CK 8 and CK 19 were targeted as they are widely expressed by mucosal epithelial tissues (Moll *et al*., 1982; Sun *et al*., 1984) and CK 20 was selected as a more discriminatory marker, particularly of cells of gastrointestinal derivation (Moll *et al*., 1992, 1993).

### Materials and methods

### *Cell lines*

The three well-characterised human cell lines used in the study were the transitional cell carcinoma-derived RT112 cell line, the breast adenocarcinoma MCF-7 cell line and the colonic adenocarcinoma HT29 cell line. MCF-7 and HT29 cells express CK 8, CK 18 and CK 19 (Moll *et al*., 1982), whereas RT112 expresses CK 8, CK 18, CK 19 along with other CK isotypes characteristic of bladder epithelial cells (Wu *et al*., 1982). CK 20, the most recently identified CK isotype, is expressed by HT29 cells, but not by MCF-7 cells (Moll *et al*., 1992). All cell lines were maintained in a 1:1 mixture of Dulbecco's modified Eagle medium (DMEM) and RPMI-1640 media supplemented with 5% fetal bovine serum and passaged using 0.25% trypsin in versene (0.02% EDTA).

### *Blood and bone marrow samples*

Normal blood or bone marrow samples were obtained from volunteers aged between 18 and 45 years. Samples were taken into EDTA, aliquoted into 2 ml volumes and frozen at −80°C until required for RNA extraction.

### *RNA extraction*

Total cellular RNA was extracted from cell lines, normal whole blood, normal bone marrow or spiked normal blood using Ultraspec RNA (Biogenesis, Bournemouth, UK) according to the manufacturer's instructions.

### *Reverse transcriptase-polymerase chain reaction (RT-PCR)*

The RT-PCR method used was based on that for the detection of neuroblastoma cells (Burchill *et al*., 1994). Briefly, dilution curves of RNA were DNAse treated and reverse transcribed to produce cDNA using a random hexamer primer. RT products were amplified by PCR for CK 8, CK 19 or CK 20 (primer sequences are given in Table I). RT-PCR products were analysed by agarose gel electrophoresis and ethidium bromide staining. Reverse transcriptase negative controls (RT −ve) in which reverse transcriptase enzyme was omitted were included for all RT-PCR reactions. Water negative controls (W) contained all components for the RT-PCR reaction but no target RNA. Where appropriate, positive controls (+C) of RNA extracted from HT29, MCF7 or RT112 cells were included. Molecular weight markers (φX 174RF DNA *Hae*III, Gibco BRL, Paisley, UK, or 123 bp ladder, Pharmacia, Milton Keynes, UK) were included on all agarose gels.

The quality of RNA was confirmed by amplification of cDNA for glyceraldehyde phosphate dehydrogenase (GAPDH) or 18S probed Northern blot analysis. All primers were purchased from Oswell DNA Services (Edinburgh, UK).

Correspondence: K Pittman, Division of Oncology, Royal Brisbane Hospital, Herston Road, Brisbane, Q 4029, Australia
Received 28 June 1994; revised 28 September 1994; accepted 30 September 1994

**Table I** Primer sequences used for PCR amplification of CK 8, CK 19 and CK 20

| | *Sense primer* | *Antisense primer* |
|---|---|---|
| CK 8 | AACAACCTTAGGCGGCAGCT | GCCTGAGGAAGTTGATCTCG |
| CK 19 | GCGGGACAAGATTCTTGGTG | CTTCAGGCCTTCGATCTGCAT |
| CK 20 | CAGACACACGGTGAACTATGG | GATCAGCTTCCACTGTTAGACG |

Primer sequences for PCR were selected using the Dieffenbach Selection Programme. Primers were located within different exons and were either 20-, 21- or 22-mers.

*Specificity of RT–PCR*

RT–PCR products were separated on agarose gels and Southern blotted onto nylon membrane (Hybond N$^+$, Amersham, UK). Filters were hybridised with a gamma $^{32}P$ end-labelled oligonucleotide probe, the sequence of which lay between each primer set. The nucleotide sequence of RT–PCR products was confirmed by dideoxy chain termination sequencing (Sequenase, USS, Canada).

*Cell spiking*

Cell spiking experiments were used to test the potential sensitivity of this technique for detection of colon carcinoma cells in blood. Known numbers of HT29 cells were added to whole blood samples, mRNA extracted and RT–PCR for CK 20 performed. To 2 ml aliquots of whole blood 10 to $1 \times 10^6$ cells were added; an unspiked blood sample was included in each experiment. RNA (100 pg) from HT29 cells was included as a positive control.

### Results

*RT–PCR detection of bladder, breast and colon carcinoma cells*

RT–PCR for CK 8 generated a single 244 bp band identified on ethidium bromide-stained agarose gels (Figure 1a). This fragment was confirmed by Southern blot analysis and sequencing (data not shown) to be the fragment of CK 8 which lies between the two primers used for PCR. The band was detected in 10 pg to 100 ng of total RNA from RT112 cells.

RT–PCR for CK 19 generated a single 214 bp band identified on ethidium bromide-stained agarose gels (Figure 1b) which was confirmed to be CK 19 by Southern blot analysis and sequencing (data not shown). This band was detected in 100 pg to 1 ng of total RNA from MCF7 cells.

RT–PCR for CK 20 geneated a single band of 370 bp (Figure 1c). This band was confirmed by Southern hybridisation and sequence analysis (data not shown) and detected in 100 pg of total RNA from HT29 cells.

In all three cases there was an increase in band intensity with increasing amounts of RNA (Figure 1). No transcripts were identified in water control samples (Figure 1) or RT-negative samples (results not shown).

*Control blood and bone marrow analysis*

In 8/9 and 6/15 control blood samples analysed CK 8 and CK 19 RT–PCR products were identified under the described conditions. Southern blotting confirmed that amplified bands were CK 8 and CK 19 RT–PCR products (results not shown). In 15/15 control blood samples analysed, CK 20 was undetectable by ethidium bromide staining or Southern blot hybridisation. RT–PCR results for CK 8 (Figure 2a), CK 19 (Figure 2b) or CK 20 (Figure 2c) are shown for six control bloods: 6/6 were positive for CK 8, 3/6 for CK 19 and 0/6 for CK 20.

RT–PCR for CK 20 in 6/6 normal bone marrow samples showed no amplified bands (Figure 3a). The integrity of bone marrow RNA samples was confirmed by RT–PCR for GAPDH (Figure 3b).

**Figure 1** Products of RT–PCR for CK 8, 19 and 20 mRNA on 1 pg to 100 ng of total mRNA isolated from RT112 (CK 8), MCF7 (CK 19) and HT29 (CK 20) cell lines. A single band of 244, 214 and 370 bp respectively was identified after separation of products in an agarose gel and staining with ethidium bromide. There was an increase in the intensity of this band with increasing RNA concentration. M, molecular weight markers; W, water control.

**Figure 2** Products of RT–PCR for CK 8 (**a**) 19 (**b**) and 20 (**c**) mRNA separated by agarose gel electrophoresis and stained with ethidium bromide in six control bloods (1–6). C, positive control for CK 8, 19 or 20 mRNA detection; W, water negative control; M, molecular weight markers.

**Figure 3** Products of RT–PCR for CK 20 mRNA separated by electrophoresis, and stained with ethidium bromide in six control bone marrow samples **a**, RT–PCR for GAPDH in the same RNA samples (**b**). +C, positive control of RNA extracted from HT29 cells; W, water negative control; M, molecular weight markers.

**Figure 4** Products of RT–PCR for CK 20 mRNA separated by agarose gel electrophoresis and stained with ethidium bromide in blood samples spiked with $1-10^4$ HT29 cells. A single 370 bp band was identified when as few as 100 cells per ml of whole blood were analysed (3). No band was identified in unspiked blood (0). RT-negative samples showed no amplified band (**b**). +C, positive control of RNA extracted from HT29 cells; W, water negative control; M, molecular weight markers.

*Cell spiking*

In HT29 cell spiking experiments it was possible to detect down to 100 HT29 cells diluted in 2 ml of whole human blood (Figure 4a). The 370 bp band generated was shown by Southern blotting to hybridise to a $^{32}P$ end-labelled oligonucleotide probe specific for CK 20 and confirmed by sequence analysis to be that of CK 20 (results not shown). No RT–PCR products were detected in whole blood alone (Figure 4a, 0). RT–PCR products were not identified in reverse transcriptase-negative samples (Figure 4b).

### Discussion

The expression of cytokeratins by epithelial tissues and their expression following neoplastic transformation had made them reliable markers in diagnostic surgical pathology. We have looked at the possible use of CKs as targets for RT–PCR detection of metastasising tumour cells of epithelial origin.

Since CK 8 and CK 19 were expressed in a high proportion of normal peripheral blood samples (88% and 40% respectively), neither would be suitable targets for detection of tumour cells in peripheral blood. These findings for CK 8 confirm the findings of Traweek *et al.* (1993), though no CK 19 expression was reported by Traweek *et al.* (1993) in bone marrow or peripheral blood. This is in contrast to the findings reported here. The number of normal peripheral blood samples analysed in this study was greater than in the study of Traweek *et al.*, which may explain the discrepancy in results. The presence of CK 19 pseudogenes (Bader *et al.*, 1986; Savtchenko *et al.*, 1988) further complicates interpretation of data using CK 19. Datta *et al.* (1994) have recently reported on the use of CK 19 for the detection of breast carcinoma micrometastasis using primer sequences selected to incorporate differences between CK 19 and pseudogene at the 3′ end. This would not exclude amplification of other CK 19 pseudogenes that do not differ at the 3′ end and would limit the value of CK 19 for this purpose.

CK 20 mRNA was not detected in any normal blood or bone marrow samples examined, suggesting it to be the CK of choice for detection of some carcinomas of epithelial origin. CK 20 has been detected in almost all cases of colorectal adenocarcinomas by immunohistochemistry (Moll *et al.*, 1987) and may prove to be a useful target for the detection of disseminating colon carcinoma by RT–PCR. To evaluate further the clinical value of CK 20 as a target gene for the detection of colorectal carcinoma metastatic cells we are examining expression of CK 20 in blood and bone marrow samples from patients.

### References

BADER BL, MAGIN TM, HATZFELD M AND FRANKE WW. (1986). Amino acid sequence and gene organisation of cytokeratin no. 19, an exceptional tail-less intermediate filament protein. *EMBO J.*, **5**, 1865–1875.

BURCHILL SA, BRADBURY FM, SMITH B, LEWIS IJ AND SELBY P. (1994). Neuroblastoma cell detection by reverse transcriptase-polymerase chain reaction (RT–PCR) for tyrosine hydroxylase mRNA. *Int. J. Cancer.*, **57**, 671–675.

COOPER D, SCHERMER A AND SUN TT. (1985). Classification of human epithelia and their neoplasms using monoclonal antibodies to keratins: strategies, applications and limitations. *Lab. Invest.*, **52**, 243–256.

DATTA YH, ADAMS PT, DROBYSKI WR, ETHIER SP, TERRY VH AND ROTH MS. (1994). Sensitive detection of occult breast cancer by the reverse transcriptase polymerase chain reaction. **12**, 475–482.

LANE EB AND ALEXANDER CM. (1990). Use of keratin antibodies in tumor diagnosis. *Sem. Cancer Biol.*, **1**, 165–179.

MOLL R, FRANKE WW, SCHILLER DL AND GEIGER B. (1982). Catalogue of human cytokeratins. Patterns of expression in normal epithelia, tumours and cultured cells. *Cell*, **31**, 11–24.

MOLL R, ROBINE S, DUDOUET B AND LOUVARD D. (1987). Villin: a cytoskeletal protein and a differentiation marker expressed in some human adenocarcinomas. *Virchows Arch B*, **54**, 155–169.

MOLL R, LOWE A, LAUFER J, FRANKE WW. (1992). Cytokeratin 20 in human carcinomas. A new histodiagnostic marker detected by monoclonal antibodies. *Am. J. Pathol.*, **140**, 427–447.

MOLL R, ZIMBELMANN R, GOLDSCHMIDT MD, KEITH M, LAUFER J, KASPER M, KOCH PJ AND FRANKE WW. (1993). The human gene encoding cytokeratin 20 and its expression during fetal development and in gastrointestinal carcinomas. *Differentiation*. **53**, 75-93.

NAGLE RB. (1988). Intermediate filaments: a review of basic biology. *Am. J. Surg. Pathol.*, **12** (Suppl. 1), 4-16.

OSBORNE M AND WEBER K. (1983). Tumour diagnosis by intermediate filament typing: A novel tool for surgical pathology. *Lab. Invest.*, **48**, 372-394.

SAVTCHENKO ES, SCHIFF TA, JIANG C-K, FREEDBERG IM AND BLUMENBERG M. (1988). Embryonic expression of the human 40-kD keratin: evidence from a processed pseudogene sequence. *Am. J. Hum. Genet.*, **43**, 630-637.

SMITH B, SELBY P, SOUTHGATE J, PITTMAN K, BRADLEY C AND BLAIR GE. (1991). Detection of melanoma cells in peripheral blood by means of reverse transcriptase and polymerase chain reaction. *Lancet*, **338**, 1227-1229.

SUN T-T, EICHNER R, SCHERMER A, COOPER D, NELSON WG AND WEISS RA. (1984). Classification, expression, and possible mechanisms of evolution of mammalian epithelial keratins: a unifying model. In *Cancer Cells: The Transformed Phenotype*. Levine AJ, Van de Woude GF, Topp WC and Watson JD (eds) pp. 169-176. Cold Spring Harbor Laboratory Press: Cold Spring Harbor, NY.

TRAWEEK ST, LIU J AND BATTIFORA H. (1993). Keratin expression in non-epithelial tissues. *Am. J. Pathol.*, **142**, 1111-1118.

WU Y-J, PARKER LM, BINDER NE, BECKETT MA, SINARD JH, GRIFFITHS CT AND RHEINWALD JG. (1982). The mesothelial keratins: a new family of cytoskeletal proteins identified in cultured mesothelial cells and nonkeratinizing epithelia. *Cell*, **31**, 693-703.